# ICU scoring systems: which one to use in oncology patients?

**DOI:** 10.1186/cc9923

**Published:** 2011-03-11

**Authors:** D Juneja, P Nasa, O Singh, R Dang, Y Javeri, G Singh

**Affiliations:** 1 Max Superspeciality Hospital, Delhi, India

## Introduction

The aim was to assess the performance of various ICU scoring systems in oncology patients.

## Methods

A prospective analysis of data for all oncology patients admitted to the ICU over 6 months. For mortality prediction, SMR was computed. Calibration was assessed by Lemeshow-Hosmer goodness-of-fit test and discrimination by AUROC curves. Primary outcome was ICU mortality.

## Results

ICU mortality was 36.5%. Mortality predicted by SAPS II score was closest to that of actual mortality with a SMR of 1.003, followed by that of MPM II0 (0.855) and APACHE II (1.181) scores (Table 1). SAPS II (X^2 ^= 1.842; *P *= 0.985) had the best calibration. Mechanical ventilation and use of vasopressors were significant baseline characteristics (Table 1). All of the scores tested had good efficacy but none reached statistical significance (Table 2).

**Table 1:** AUC for predicting ICU mortality

Parameter	Survivors	Nonsurvivors	*P *value
Males	44	26	0.854
Females	22	12	
Metastasis	42	22	0.562
Ventilation	5	35	0.00
Vasopressors	7	37	0.00

**Table 2:** Baseline characteristics of survivors and nonsurvivors

Scoring system	AUC	95% CI
APACHE II	0.726	0.629 to 0.824
APACHE III	0.818	0.733 to 0.903
APACHE IV	0.793	0.707 to 0.880
SAPS II	0.718	0.615 to 0.820
SAPS III	0.781	0.686 to 0.877
MPM II_0_	0.750	0.648 to 0.853
MPM III_0_	0.684	0.573 to 0.795
SOFA	0.769	0.678 to 0.859

## Conclusions

The SAPS II and APACHE III scores showed good accuracy, calibration and mortality prediction. Nevertheless, the difference in efficacy was not statistically significant and the choice of scoring system may depend on the ease of use and local preferences.

